# Functions and mechanisms of lncRNA MALAT1 in cancer chemotherapy resistance

**DOI:** 10.1186/s40364-023-00467-8

**Published:** 2023-02-24

**Authors:** Junhui Hou, Gong Zhang, Xia Wang, Yuan Wang, Kefeng Wang

**Affiliations:** 1 Department of Urology, Shengjing Hospital of China Medical University, #36 Sanhao Street, Heping District, Shenyang, 110004 Liaoning China; 2 Department of General Surgery, Shengjing Hospital of China Medical University, #36 Sanhao Street, Heping District, Shenyang, 110004 Liaoning China

**Keywords:** Long non-coding RNA, MALAT1, Chemotherapy resistance, Cancer, Treatment

## Abstract

Chemotherapy is one of the most important treatments for cancer therapy. However, chemotherapy resistance is a big challenge in cancer treatment. Due to chemotherapy resistance, drugs become less effective or no longer effective at all. In recent years, long non-coding RNA metastasis-associated lung adenocarcinoma transcript 1 (MALAT1) has been found to be associated with the development of chemotherapy resistance, suggesting that MALAT1 may be an important target to overcome chemotherapy resistance. In this review, we introduced the main mechanisms of chemotherapy resistance associated with MALAT1, which may provide new approaches for cancer treatment.

## Introduction

Over the past few decades, chemotherapy, as a supplement to surgical treatment, has become the treatment of choice for patients with advanced cancer, reducing tumor residue and preventing tumor recurrence. Numerous studies have shown that combined chemotherapy can significantly improve the prognosis of tumor patients [1, 2]. According to its cytotoxic mechanism, chemotherapy can be divided into the following six categories: (1) alkylation agents, such as cyclophosphamide; (2) anti-metabolites, such as 5-fluorouracil (5-FU) and methotrexate, which can inhibit DNA synthesis; (3) antibiotics, such as doxorubicin and mitomycin C; (4) DNA topoisomerase inhibitors, such as etoposide, which can prohibit transcription and replication; (5) mitosis inhibitors, such as paclitaxel and vincristine (VCR); (6) platinum-based drugs, such as cisplatin (DDP) and oxaliplatin (OXA), which can induce DNA-platinum adducts to block DNA repair [3–6]. Chemotherapy affects the cell cycle by inducing DNA breakdown and blocking DNA repair, which in turn leads to tumor cell death. Although chemotherapy is effective, intrinsic and acquired chemotherapy resistance remains a huge challenge in cancer therapy.

**Fig. 1:**
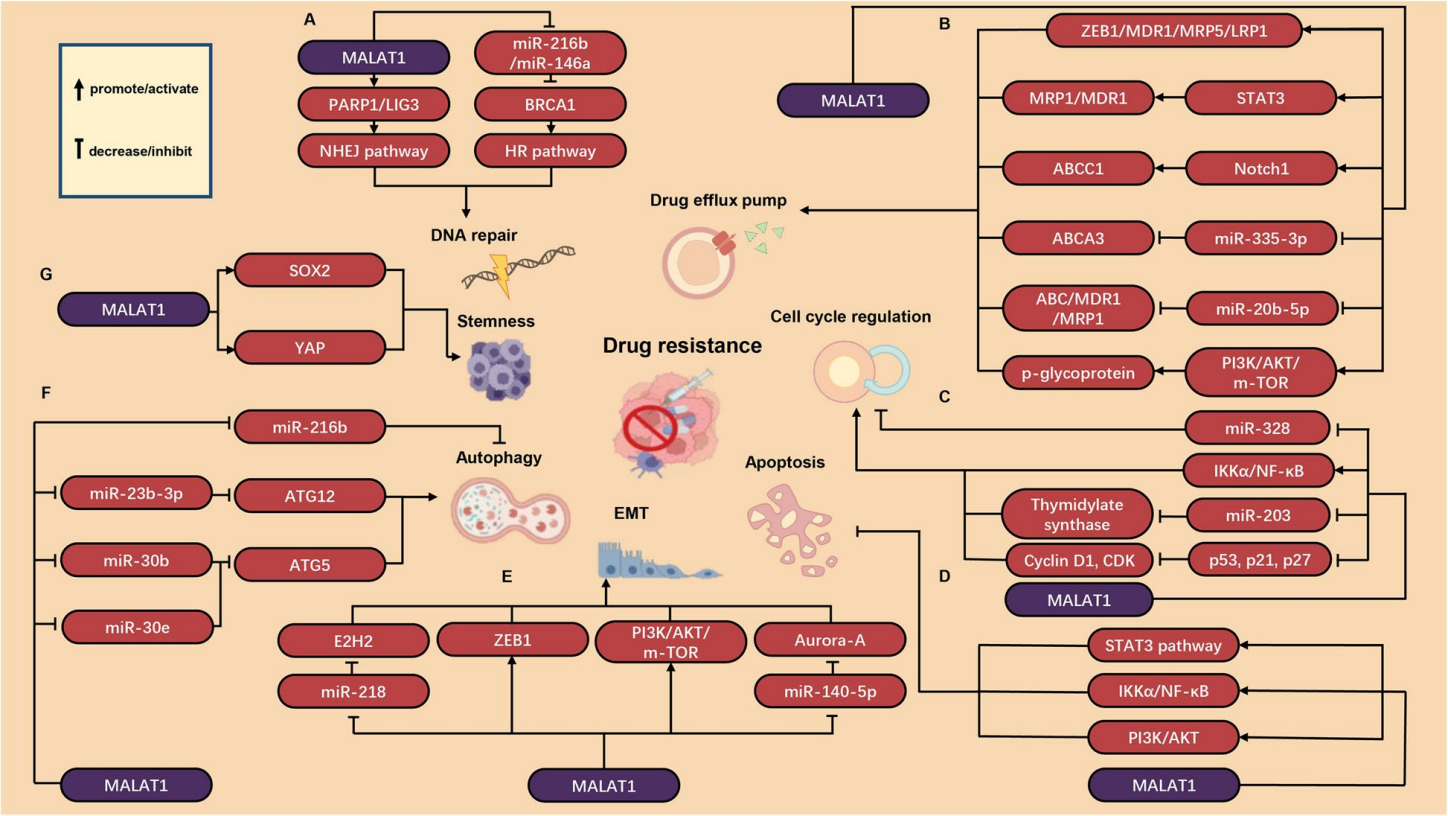
Molecular mechanisms of MALAT1 in chemotherapy resistance. Molecular mechanisms of (**A**) DNA repair pathway, (**B**) drug efflux pump regulation, (**C**) cell cycle regulation, (**D**) apoptosis regulation, (**E**) EMT promoting, (**F**) autophagy regulation, and (**G**) stemness

Chemotherapy resistance is a condition in which a disease becomes resistant to chemotherapy drugs. Once chemotherapeutic resistance occurs, the drug becomes less effective or no longer effective at all. For example, the recurrence rate of ovarian cancer (OC) increased to 70% due to DDP resistance, resulting in a 5-year survival rate of less than 50% [7]. The mechanism of chemotherapy resistance in cancer can be explained as follows: (1) increasing the ability of DNA damage repair; (2) influencing drug transport and metabolism, and affecting drug kinetics; (3) evading cell cycle checkpoint; (4) inhibiting cells apoptosis, and protecting cells from death; (5) promoting epithelial-mesenchymal transition (EMT); (6) altering the autophagy system of tumor cells; (7) modulating properties of cancer stem cells (Fig. 1). However, the exact mechanism remains to be studied.

Previous studies have focused on the mechanism of protein-coding genes in cancer chemotherapy resistance [8, 9]. However, protein-coding genes account for only about 2% of the human genome and most of the rest are untranslated genes, which has attracted wide attention in recent years. The transcription products of untranslated genes are called non-coding RNAs (ncRNAs), and long ncRNAs (lncRNAs) are one of them. LncRNAs are greater than 200 nucleotides in length and are initially thought to be transcriptional noise. However, emerging evidence suggests that lncRNAs play important roles in chemotherapy resistance of cancer [10–13].

**Fig. 2:**
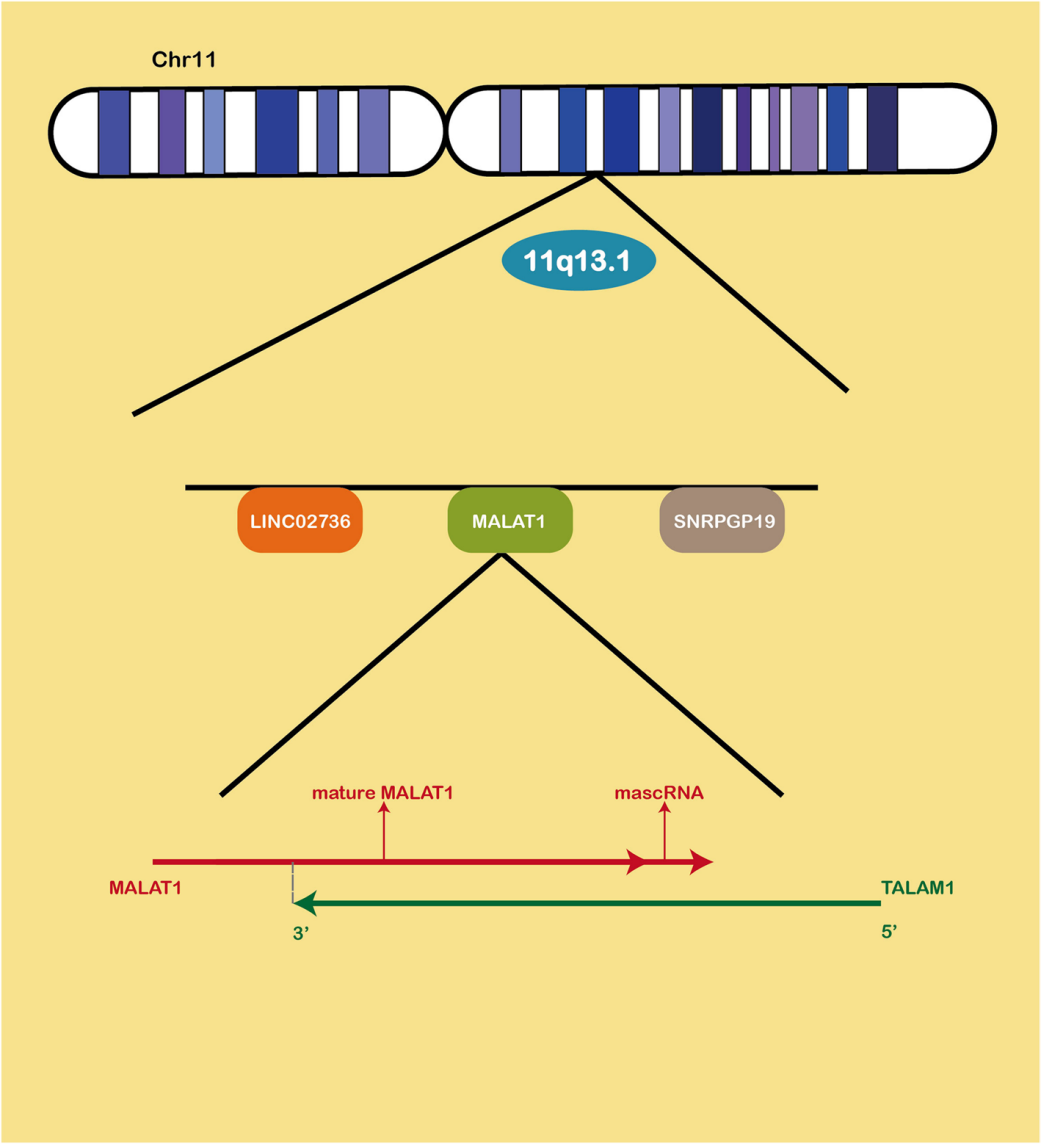
Schematic location of MALAT1. MALAT1 is located at 11q13.1, between LINC02736 and SNRPGP19. Its transcription products include MALAT1, mascRNA, and TALAM1 (the natural antisense RNA of MALAT1)

MALAT1 was first identified in non-small cell lung cancer (NSCLC) patients and was upregulated in tumors with a high metastatic tendency [14]. MALAT1 gene is encoded on human chromosome 11q13.1, which has a high evolutionary conservation [15]. MALAT1 is approximately 8.7 knt and is transcribed by RNA polymerase II (Pol II) (Fig. 2) [15]. Abnormal expression of MALAT1 has been reported to be associated with the occurrence, progression, metastasis and chemotherapy resistance of cancers [16–19]. MALAT1 has been shown to be associated with various human diseases, including neoplastic and non-neoplastic diseases, such as oral squamous cell carcinoma, psoriasis, recurrent miscarriage, major adverse cardiac and cervical events [20–24]. Moreover, MALAT1 has been found to be a potential therapeutic target for cancer. For example, a recent study revealed that targeting the MALAT1/PARP1 (poly ADP-ribose polymerase 1) / LIG3 (DNA Ligase 3) complex resulted in DNA damage and apoptosis in multiple myeloma [25]. In addition, it has been proved that targeting MALAT1 also played a significant role in the treatment of gynecological cancer [26]. Previous studies have shown that MALAT1 affected cancer through multiple mechanisms. Therefore, we suspect that MALAT1 also plays a crucial role in chemotherapy resistance.

A large number of studies have found that there exists a high correlation between MALAT1 and chemotherapy resistance. However, there are few reviews on MALAT1 and chemotherapy resistance. Therefore, it is necessary to investigate the mechanism of MALAT1 in chemotherapy resistance to explore new tumor therapeutic targets.

In this review, we discussed the functions and mechanisms of MALAT1 in cancer chemotherapy resistance.

## Biogenesis and functions of MALAT1

### Biogenesis of MALAT1

**Fig. 3:**
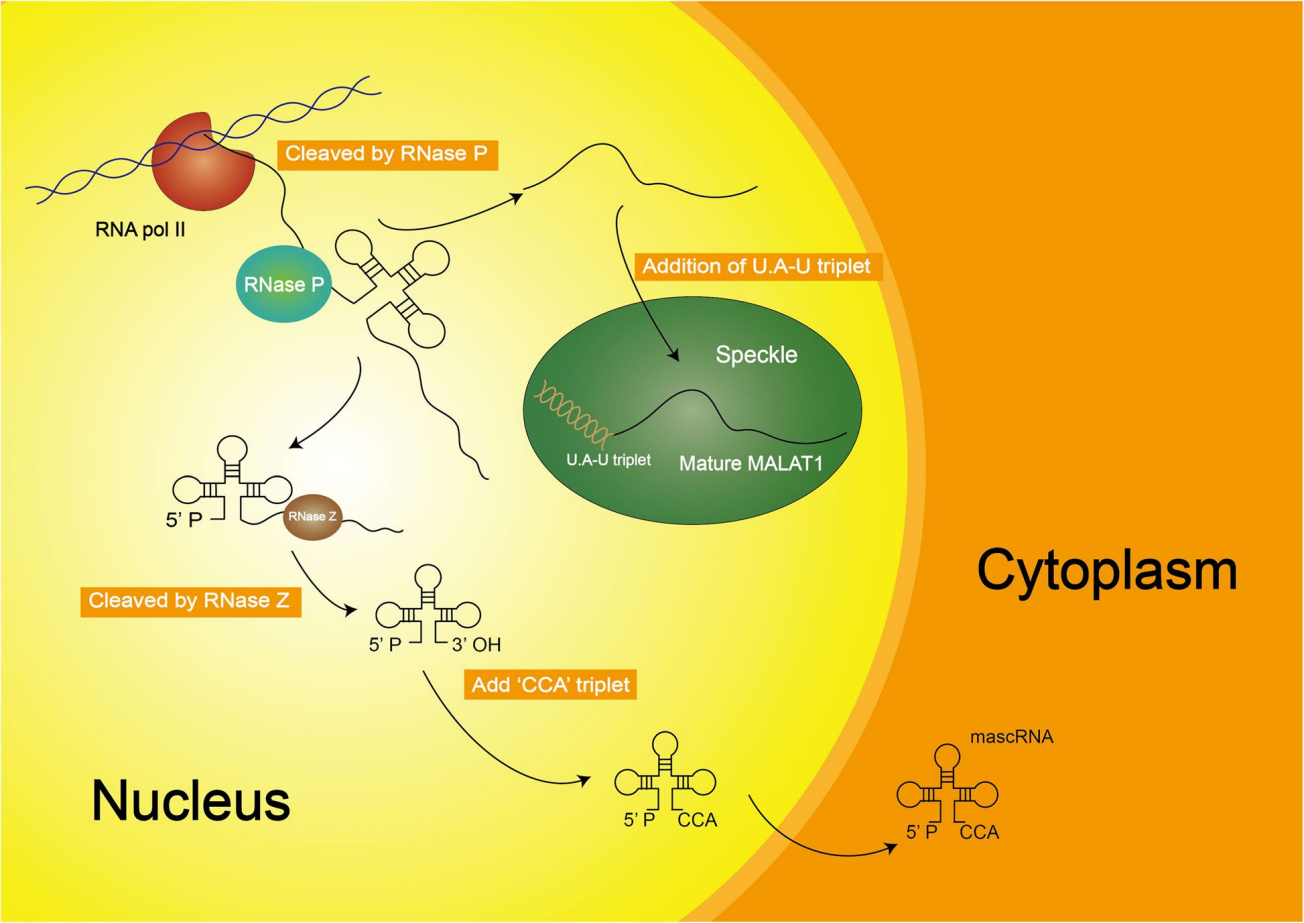
Biogenesis of MALAT1. The original MALAT1 is transcribed by RNA Pol II, and then cleaved by RNase P to form mature MALAT1 and a smaller RNA. The mature MALAT1 localizes to the nuclear speckles, while the smaller RNA is further processed into mascRNA by RNase Z and CCA-adding enzymes and transported into the cytoplasm

MALAT1, a ubiquitously expressed gene, is located on human chromosome 11q13 and mouse chromosome 19qA [27, 28]. Furthermore, the expression level of MALAT1 is quite high, even comparable to several protein-coding genes such as GADPH [28]. MALAT1 is similar to mRNA that encodes the protein and is synthesized by RNA Pol II. The transcript of MALAT1 is about 7 kb in human and 6.7 kb in mice [29, 30]. MALAT1 lacks a poly(A) tail at the 3' end, which differs from the typical cleavage and polyadenylation mechanism [31]. Instead, ribonuclease (RNase) P cuts the original transcript of MALAT1 into a mature 7 kb transcript, and into a much smaller transcription fragment at the 3’ end [29–32]. The RNase P cleaves MALAT1 at nt7518 to produce the 5' end of small RNA and the 3' end of mature MALAT1. The 3' end of mature MALAT1 is a highly conserved triple helix structure, distinct from the conventional poly (A) tail. It is composed of one A-rich tract and two U-rich motifs encoded by the genome [33]. Due to the unique triple helix structure at its 3’ end, MALAT1 has high stability and is not easy to cut [34]. Moreover, the natural antisense transcript TALAM1 also promotes the stability of MALAT1 through a feedforward positive regulatory loop [35]. Unlike other Pol II-produced RNAs, which are transported to the cytoplasm for further processing immediately after transcription, mature MALAT1 transcripts are enriched in nuclear speckles in human and mice [30, 36]. The location of MALAT1 suggests that MALAT1 is involved in physiological and pathological processes [37, 38]. However, knockdown of MALAT1 in mice does not cause phenotypic changes [34]. The possible reason is that MALAT1 has no obvious effect under normal conditions. Short fragments are cleaved and processed by RNase Z and CCA-adding enzymes to form a 61-nt-lncRNA, called MALAT1-associated small cytoplasmic RNA (mascRNA), which is then folded into a tRNA-like cloverleaf structure and transported into the cytoplasm (Fig. 3) [31]. However, the functions and effects of mascRNA remain to be further studied.

### Biological functions of MALAT1

Collecting studies have found that MALAT1 can regulate biological function through intermolecular interactions. (1) Regulating gene transcription. In multiple myeloma (MM), the transcription factor Sp1 can be recruited by MALAT1 to promote the secretion of TGF-β by binding to latent transforming growth factor-β binding protein-3 [39]. (2) Regulating RNA splicing. MALAT1 can interact with the splicing factors of serine- and arginine-rich splicing factor (SRSF) 1, SRSF2, SRSF3 and other SR proteins, affect the distribution of splicing factors in the nuclear speckle domains, and regulate the alternative splicing of pre-mRNAs [40]. (3) Regulating protein activity. MALAT1, as a splicing factor proline-and glutamine-rich (SFPQ) bound competitor, will accelerate the dissociation of PTBP2 from the SFPQ/PTBP2 complex, enhance the function of PTBP2, and promote the proliferation and migration of tumor cells [41]. (4) Regulating epigenetic change. MALAT1 can recruit the suppressor of variegation 3–9 homolog 1 to MyoD-binding loci and cause the trimethylation of histone 3 lysine 9, which suppresses the transcriptional activity of MyoD [42]. (5) Regulating the nuclear and cytoplasmic transport of proteins. During cell division, MALAT1 alters the transport of the nucleus to the cytoplasm by binding to an abundant nuclear factor heterogeneous nuclear ribonucleoprotein C protein [43]. (6) Acting as a competitive endogenous RNA (ceRNA). Collecting studies have found that MALAT1 alters a series of life activities by acting as a ceRNA [44–46]*.* MALAT1 competitively sequestered miR-23b-3p and attenuated the inhibitory effect of miR-23b-3p on ATG12, thereby increasing the expression of ATG12 and promoting autophagy associated chemotherapy resistance of gastric cancer (GC) cells [44]. Moreover, MALAT1 can act as a ceRNA to inhibit miR-181c-5p, leading to berberine mediated inhibition of HMGB1 in poststroke inflammation [45]. MALAT1 regulated mTOR-mediated Tau hyperphosphorylation by acting as a ceRNA sponge of miR-144 in hippocampal cells [46]. Because MALAT1 extensively affects life activities and cell phenotypes through the above multiple pathways, it is possible that MALAT1 affects tumor chemotherapy resistance through the above mechanisms.

## Mechanisms mediating chemotherapy resistance related to MALAT1 in cancers

### MALAT1 associated with DNA damage repair pathway

To maintain genomic stability, normal cells can repair DNA damage caused by internal oxidative stress, external radiation, and cytotoxic drugs. If normal processes are blocked, the genome of cells may become unstable, leading to the development of tumors. There are two main molecular pathways to repair damaged DNA: homologous recombination (HR) pathway and non-homologous end junction (NHEJ) pathway [47, 48]. However, some cancer cells evade chemotherapy by enhancing their DNA repair abilities, leading to chemotherapy resistance [49].

Recent studies have shown that MALAT1 affected many factors involved in DNA repair (Fig. 1A, Table 1). In MM, MALAT1 enhanced the NHEJ pathway by binding to PARP1 and LIG3, two key molecules in this pathway. Researchers found that anti-MALAT1 induced DNA damage and reduced drug resistance to bortezomib, melphalan, and doxorubicin, suggesting that MALAT1 may induce the development of resistance by enhancing DNA damage repair [25]. MALAT1 is not only involved in the NHEJ pathway, but also the HR pathway. In NSCLC, researchers found that targeting MALAT1 could induce DNA damage even when the NHEJ pathway was blocked. MALAT1 sponged miR-146a and miR-216b to protect BRCA1, functioning as a ceRNA. As BRCA1 was a key upregulation factor in the HR pathway, upregulation of BRCA1 expression led to enhancement of the HR pathway. Finally, MALAT1 overexpression resulted in DDP resistance in NSCLC cells [50].

**Table 1:** MALAT1 associated with DNA damage repair pathway

Cancer type	Expression	Related drugs	Target	Related genes or pathway	References
Myeloma	upregulation	bortezomib, melphalan and doxorubicin	/	PARP1/LIG3	[25]
Lung cancer	upregulation	cisplatin	miR-146a and miR-216b	BRCA1	[50]

In all, abnormally high expression of MALAT1 enhances the DNA repair ability of cancer cells through the above pathways, leading to drug resistance.

### MALAT1 involved in drug efflux pump function

Reducing intracellular drug concentration is a major and direct way to obtain chemotherapy resistance in tumor cells, which is associated with drug efflux system, such as ATP-binding cassette (ABC) membrane transporter proteins [19, 20]. ABC proteins can affect pharmacokinetics by altering the transport of various drugs. Therefore, the overexpression of ABC protein is the main cause of multidrug resistance. Multidrug resistant protein 1 (MDR1), multidrug resistance like protein 1 (ABCC1 or MRP1), multidrug resistance associated protein 2, and ABC subfamily G2 are relatively common and well-known ABC proteins [51–53].

A large number of studies have shown that MALAT1 altered drug distribution by regulating the ABC proteins (Fig. 1B, Table 2). In glioblastoma, si-MALAT1 downregulated the expression of ZEB1, MDR1, MRP5 and LRP1, enhancing the sensitivity to Temozolomide (TMZ) [54, 55]. Conversely, overexpression of MALAT1 led to resistance to TMZ. However, the exact mechanism by which MALAT1 affects the expression of these proteins is still being investigated. It was found that overexpression of MALAT1 upregulated the expression of MRP1 and MDR1 by activating STAT3, thus promoting DDP resistance in NSCLC [56]. Another study pointed out that MALAT1 upregulated ABCC1 expression by activating Notch1, enhancing DDP resistance in OC cells [57]. In addition, MALAT1 was found to be involved in ABCA3 regulation by downregulating miR-335-3p in the multidrug resistant group of childhood acute lymphoblastic leukemia, which may lead to chemotherapy resistance [58]. In colorectal cancer (CRC), suppression of MALAT1 restrained cell progression by downregulation of the expression of ABC, MDR1, MRP1, and breast cancer drug resistant proteins through targeting miR-20b-5p, resulting in drug resistance of cancer cells to 5-FU [59]. In oral squamous cell carcinoma (OSCC), MALAT1 developed the DDP resistance of OSCC by upregulating P-glycoprotein expression [60].

**Table 2:** MALAT1 involved in drug efflux pump function

Cancer type	Expression	Related drugs	Target	Related genes or pathway	References
Glioma	upregulation	temozolomide	/	ZEB1	[54]
Glioma	upregulation	temozolomide	/	p53	[55]
Lung cancer	upregulation	cisplatin	/	STAT3/MRP1 and MDR1	[56]
Ovarian cancer	upregulation	cisplatin	/	Notch1	[57]
Leukemia	upregulation	multidrug resistance	miR-335-3p	ABCA3	[58]
Colorectal cancer	upregulation	5-fluorouracil	miR-20b-5p	ABC, BCRP, MDR1, and MRP1	[59]
Oral squamous carcinoma	upregulation	cisplatin	/	PI3K/AKT/m-TOR	[60]

Through these pathways, MALAT1 enhances the function of drug efflux pump in tumor cells, reduces intracellular drug concentration, and leads to drug resistance of tumors.

### MALAT1 involved in cell cycle regulation

To ensure the quality and integrity of DNA replication, there is a mechanism in the cell cycle to solve the abnormal events that occur during DNA replication, which is known as the cell cycle checkpoint. The cell cycle checkpoint system consists of many molecules, including upstream protein kinases such as Ataxia telangiectasia-mutated and ataxia telangiectasis and Rad3 related, and downstream checkpoint proteins such as CHK1, CHK2, BRCA, and p53 [61, 62]. Cancer cell resistance to chemotherapy may be due to defects in factors associated with cell cycle checkpoints [63].

There is growing evidence that MALAT1 has a regulatory role in the cell cycle that reduces the sensitivity to chemotherapy (Fig. 1C, Table 3). For instance, it was found that MALAT1 promoted TMZ resistance in glioblastoma multiforme (GBM) cells. MALAT1 promoting the thymidylate synthase by downregulating miR-203, resulting in TMZ resistance, which was demonstrated by an increased percentage of cell population in G0/G1 phase [64]. Moreover, suppression of MALAT1 downregulated the expression of Cyclin D1 and CDK, and upregulated the expression of p53, p21, and p27, resulting in an increase of sensitivity of hepatocellular carcinoma (HCC) cells to 5-FU [65]. In addition, MALAT1 silencing greatly blocked the cell cycle of chronic myeloid leukemia (CML) cells and inhibited cell proliferation by releasing the sponging effect on miR-328, which attenuated the chemotherapy resistance of CML cells to imatinib [66]. Another study pointed out that MALAT1 was overexpressed in head and neck squamous cell carcinoma (HNSCC) cells. Downregulation of MALAT1 resulted in cell cycle arrest in G(2)/M phase and enhanced sensitivity of HNSCC cells to DDP [67].

**Table 3:** MALAT1 involved in cell cycle regulation

Cancer type	Expression	Related drugs	Target	Related genes or pathway	References
Glioma	upregulation	temozolomide	miR-203	thymidylate synthase	[64]
Hepatocellular cancer	upregulation	5-fluorouracil	/	IKKα/NF-κB	[65]
Leukemia	upregulation	imatinib	miR-328	/	[66]
Head and Neck Squamous Cell Carcinoma	upregulation	cisplatin	/	/	[67]

In conclusion, the abnormal increase of MALAT1 can lead to the dysregulation of cell cycle, and then lead to the generation of drug resistance.

### Apoptosis-related MALAT1 regulating chemosensitivity

Apoptosis is a complex programmed cell death process that can be triggered by caspase-mediated external or internal pathways, involving a variety of signaling pathways. Besides, the caspase-cascade system plays a crucial role in the induction, transduction, and amplification of intracellular apoptotic signals [68]. Moreover, caspases are also regulated by numerous molecules and pathways, influencing the apoptosis. Through the action of these regulators, apoptosis will be affected, and the cell phenotypes (such as chemotherapy resistance) will be altered accordingly [69].

Recent studies have fully revealed that MALAT1 was related to apoptosis and may lead to chemotherapy resistance (Fig. 1D, Table 4) [70]. For example, in NSCLC, polyphyllin I inhibited the expression of MALAT1, resulting in the inactivation of STAT3 signaling pathway and apoptosis in gefitinib-resistant cancer cells [71]. Another research group reported that inhibition of MALAT1 had also been found to alter apoptosis through the IKKα/NF-κB pathway, thereby boosting the sensitivity of cancer cells to 5-FU in HCC [65]. In cervical cancer, overexpression of MALAT1 was found to upregulate the expression of p-PI3K, p-AKT, and BRWD1, promoting DDP resistance [72]. Similarly, in GC cells, overexpression of MALAT1 can upregulate p-PI3K, p-AKT and p-STAT3, which also contributed to DDP resistance by altering apoptosis [73].

**Table 4:** MALAT1 associated with chemosensitivity by altering apoptosis

Cancer type	Expression	Related drugs	Target	Related genes or pathway	References
Lung cancer	upregulation	gefitinib	/	STAT3	[71]
Hepatocellular cancer	upregulation	5-fluorouracil	/	IKKα/NF-κB	[65]
Cervical cancer	upregulation	cisplatin	/	PI3K/AKT	[72]
Gastric cancer	upregulation	cisplatin	/	PI3K/AKT	[73]

In general, the high expression of MALAT1 regulates apoptosis-related genes and molecules, affects cancer cell apoptosis, and triggers cancer drug resistance.

### MALAT1 associated with EMT-related chemosensitivity

EMT is a process that alters the transform of polarized epithelial cells to motile mesenchymal cells characterized by the loss of E-cadherin through the activation of one or several factors such as SNAIL, SLUG, ZEBs, and TWIST [74, 75]. During EMT, epithelial cells lost their epithelial phenotype such as cell polarity and connection to the basement membrane and acquired a mesenchymal phenotype such as higher migration and invasion, resistance to apoptosis and degradation of the extracellular matrix. EMT is therefore a mechanism for tumor metastasis. In addition, recent studies have shown that multiple factors in EMT play an important role in the development of chemotherapy resistance.

Studies have shown that MALAT1 regulated EMT, thereby promoting EMT-induced chemotherapeutic resistance (Fig. 1E, Table 5). In CRC, MALAT1 knockdown enhanced E-cadherin expression and inhibited OXA-induced EMT, which may be a promising therapeutic target for CRC patients [76]. Li et al. [54] demonstrated that EMT and MALAT1 overexpression were associated with TMZ in drug-resistant GBM cells, suggesting that MALAT1 was involved in EMT-induced chemotherapy resistance. MALAT1 regulated EMT by upregulating ZEB1, making GBM cells resistant to TMZ. In OSCC, MALAT1 expression was higher in DDP-resistant cells. Further evidence suggested that MALAT1 was involved in EMT process through upregulation of P-gp and activation of PI3K/AKT/m-TOR signaling pathway, leading to DDP resistance [60]. In HCC, MALAT1 interacted with miR-140-5p to enhance the expression of Aurora-A, leading to EMT and the formation of chemotherapy resistance to sorafenib [77].

**Table 5:** MALAT1 associated with chemosensitivity via regulating EMT

Cancer type	Expression	Related drugs	Target	Related genes or pathway	References
Colorectal cancer	upregulation	oxaliplatin	miR-218	EZH2	[76]
Glioma	upregulation	temozolomide	/	ZEB1	[54]
Oral squamous cell carcinoma	upregulation	cisplatin	/	PI3K/AKT/m-TOR	[60]
Hepatocellular cancer	upregulation	sorafenib	miR-140-5p	Aurora-A	[77]

Therefore, MALAT1 can also induce drug resistance of cancer cells by promoting the EMT.

### Autophagy-related MALAT1 associated with chemosensitivity

Autophagy is a basic process of degradation and reuse of cell components that is highly conserved in all eukaryotes. Autophagy is not only a "recycling" biological function, but also affects the response to infection, embryonic development and cellular variation, and directly affects the occurrence and development of tumors, as well as drug response and drug resistance [78–80]. The primary purpose of autophagy is to stabilize the intracellular environment. In normal cells, autophagy can reduce the risk of cancer. Paradoxically, autophagy is an important cause of chemotherapy failure in cancer cells [81].

In recent years, more and more studies have found that MALAT1 was strongly related to the regulation of autophagy in cancer cells (Fig. 1F, Table 6). In HCC, MALAT1 played a key role in the development of chemotherapy resistance by regulating autophagy. HIF-2α upregulated MALAT1 expression, which can act as a ceRNA of miR-216b to regulate autophagy, leading to 5-FU resistance [82]. Similarly, MALAT1 affected autophagy of GC cells through a variety of pathways, leading to tumor drug resistance. Hu et al. [44] revealed that MALAT1 targeted miR-23b-3p and reduced its inhibition of autophagy related gene (ATG) 12, leading to autophagy and chemotherapy resistance to 5-FU, DDP and VCR in GC. Another study pointed out that MALAT1 induced chemotherapy resistance of GC cells to DDP by inhibiting miR-30b and promoting ATG5 expression [83]. Moreover, it has been reported that propofol promotes DDP sensitivity by inhibiting autophagy in GC through MALAT1/miR-30e/ATG5 axis, suggesting that MALAT1 induced autophagy-associated chemotherapy resistance of GC cells to DDP [84].

**Table 6:** MALAT1 associated with chemosensitivity via regulating autophagy

Cancer type	Expression	Related drugs	Target	Related genes or pathway	References
Hepatocellular cancer	upregulation	5-fluorouracil	miR-216b	/	[82]
Gastric cancer	upregulation	5-fluorouracil, cisplatin, vincristine	miR-23b-3p	ATG12	[44]
Gastric cancer	upregulation	cisplatin	miR-30b	ATG5	[83]
Gastric cancer	upregulation	cisplatin	miR-30e	ATG5	[84]

In summary, MALAT1 overexpression affects the autophagy of cancer cells and leads to drug resistance.

### MALAT1 involved in the stemness-related chemosensitivity

Cancer stemness is the phenotype like normal stem cells such as plasticity and self-renewal ability, and the cancer cells with these properties are known as cancer stem cells, which is recognized to be one of the important causes of chemotherapy resistance [85, 86].

Recent studies have shown that MALAT1 was involved in the cancer stemness (Fig. 1G, Table 7). In GC cells, MALAT1 acted as a stabilizer of SOX2 mRNA by binding to it directly, resulting in stemness and chemotherapy resistance to DDP [87]. In esophageal squamous cell carcinoma, MALAT1 directly bound to Yes-associated protein (YAP) and enhanced the transcription and expression of YAP, developing the stemness and resistance to DDP [88]. In OC, MALAT1 acted as a co-activator of YAP, inhibiting its translocation, promoting the expression of YAP and thereby enhancing the effect of YAP, which contributes to the stemness and chemotherapy resistance in OC to DDP [89].

**Table 7:** MALAT1 associated with chemosensitivity via regulating stemness

Cancer type	Expression	Related drugs	Target	Related genes or pathway	References
Gastric cancer	upregulation	cisplatin	/	SOX2	[87]
Esophageal cancer	upregulation	cisplatin	/	YAP	[88]
Ovarian cancer	upregulation	cisplatin	/	YAP	[89]

As described above, MALAT1 can also contribute to cancer resistance to chemotherapeutic drugs by enhancing the stemness of cancer cells.

## Conclusion and future perspectives

With the use of chemotherapy, the prognosis of cancer patients has improved dramatically, which is a huge advancement in cancer treatment. However, the development of chemotherapy resistance is becoming an important reason for chemotherapy failure. At present, lncRNAs have been proved to be important regulatory factors involved in numerous life activities. LncRNAs are specific in many respects and are equally expressed in terms of chemotherapy resistance. MALAT1, which has been studied the most, plays an important regulatory role in tumor development. MALAT1 has been a molecule of interest since its discovery as a predictive biomarker for lung cancer metastasis [90]. Current studies show that MALAT1 is a potential target not only for cancer therapy, but also for overcoming cancer resistance. Therefore, targeting MALAT1 treatment may not only be effective against tumor therapy itself, but also make chemotherapy more effective, which may contribute to the complex therapy of cancers and improve the prognosis of cancers. As an important adjuvant to chemotherapy, immunotherapy has developed rapidly in recent years. Chemotherapy combined with immunotherapy has been widely used, and MALAT1 has been found to play an important regulatory role in immunotherapy. For instance, MALAT1 inhibited the immune response to cancer by enhancing immune escape and immunosuppressive effects, potentially leading to failure of immunotherapy and poor prognosis [91, 92]. Therefore, it is urgent to explore the mechanism and influence of MALAT1 in immunotherapy, which may provide new targets and approaches for cancer therapy. In addition, some lncRNAs can mediate chemoresistance through immune pathways. For example, lncRNA PCAT-1 can induce KRAS-related chemoresistance through immunosuppression [93]. Although there is no conclusive evidence, based on the widespread expression of MALAT1 in the immune system and its multiple effects, we speculate that MALAT1 may also influence chemotherapy resistance by affecting the immune system. However, research on the effect of MALAT1 in the chemotherapy resistance and immunotherapy of cancers is still at the nascent stage. Substantial basic research and clinical trials are needed before these molecular approaches can be applied to the clinic. Due to the extensive regulatory roles of MALAT1, it will provide new targets for cancer prevention and treatment in the future.

## Data Availability

Not applicable.

## Abbreviations

MALAT1: Metastasis-associated lung adenocarcinoma transcript 1
5-FU: 5-Fluorouracil
VCR: Vincristine
DDP: Cisplatin
OXA: Oxaliplatin
OC: Ovarian cancer
EMT: Epithelial-mesenchymal transition
NcRNAs: Non-coding RNAs
LncRNAs: Long non-coding RNAs
NSCLC: Non-small cell lung cancer
Pol II: Polymerase II
RNase: Ribonuclease
PARP1: Poly ADP-ribose polymerase 1
LIG3: DNA Ligase 3
HMGB1: High-mobility group box 1
MascRNA: MALAT1-associated small cytoplasmic RNA
MM: Multiple myeloma
SRSF: Serine- and arginine-rich splicing factor
SFPQ: Splicing factor proline-and glutamine-rich
HR: Homologous recombination
NHEJ: Non-homologous end junction
CeRNA: Competing endogenous RNA
ABC: ATP-binding cassette
MDR1: Multidrug resistant protein 1
ABCC1 or MRP1: Multidrug resistance like protein 1
TMZ: Temozolomide
CRC: Colorectal cancer
OSCC: Oral squamous cell carcinoma
GBM: Glioblastoma multiforme
HCC: Hepatocellular carcinoma
CML: Chronic myeloid leukemia
HNSCC: Head and neck squamous cell carcinoma
GC: Gastric cancer
ATG: Autophagy related gene
YAP: Yes-associated protein
